# Changing perceptions of hunger on a high nutrient density diet

**DOI:** 10.1186/1475-2891-9-51

**Published:** 2010-11-07

**Authors:** Joel Fuhrman, Barbara Sarter, Dale Glaser, Steve Acocella

**Affiliations:** 1 Hahn School of Nursing, University of San Diego, San Diego, CA, USA; 2 Southern California University of Health Sciences, Whittier, CA, USA; 3 4 Walter E. Foran Blvd, Suite 409, Flemington, NJ, USA

## Abstract

**Background:**

People overeat because their hunger directs them to consume more calories than they require. The purpose of this study was to analyze the changes in experience and perception of hunger before and after participants shifted from their previous usual diet to a high nutrient density diet.

**Methods:**

This was a descriptive study conducted with 768 participants primarily living in the United States who had changed their dietary habits from a low micronutrient to a high micronutrient diet. Participants completed a survey rating various dimensions of hunger (physical symptoms, emotional symptoms, and location) when on their previous usual diet versus the high micronutrient density diet. Statistical analysis was conducted using non-parametric tests.

**Results:**

Highly significant differences were found between the two diets in relation to all physical and emotional symptoms as well as the location of hunger. Hunger was not an unpleasant experience while on the high nutrient density diet, was well tolerated and occurred with less frequency even when meals were skipped. Nearly 80% of respondents reported that their experience of hunger had changed since starting the high nutrient density diet, with 51% reporting a dramatic or complete change in their experience of hunger.

**Conclusions:**

A high micronutrient density diet mitigates the unpleasant aspects of the experience of hunger even though it is lower in calories. Hunger is one of the major impediments to successful weight loss. Our findings suggest that it is not simply the caloric content, but more importantly, the micronutrient density of a diet that influences the experience of hunger. It appears that a high nutrient density diet, after an initial phase of adjustment during which a person experiences "toxic hunger" due to withdrawal from pro-inflammatory foods, can result in a sustainable eating pattern that leads to weight loss and improved health. A high nutrient density diet provides benefits for long-term health as well as weight loss. Because our findings have important implications in the global effort to control rates of obesity and related chronic diseases, further studies are needed to confirm these preliminary results.

## Introduction

One of the common barriers to weight loss is the uncomfortable sensation of hunger that drives overeating and makes dieting fail, even in those who are obese from over-consumption of calories. Over the past two decades we have worked closely with approximately twenty thousand patients in a private suburban family practice in New Jersey specializing in nutritional interventions for weight loss and disease prevention/management. Our experience is that enhancing the micronutrient quality of the diet even in the context of a substantially lower caloric intake dramatically mitigates the experience of hunger. A diet high in micronutrients appears to decrease food cravings and overeating behaviors. Sensations such as fatigue, weakness, stomach cramps, tremors, irritability and headaches, commonly interpreted as "hunger", resolve gradually for the majority of people who adopt a high nutrient density diet, and a new, less distressing, sensation (which we label "true" or "throat" hunger) replaces it.

It is well documented that a diet low in antioxidant and phytochemical micronutrients leads to heightened oxidative stress and a build-up of toxic metabolites [1-4]. It has also been shown that a higher intake of nutrient rich plant foods decreases measurable inflammatory by-products [5-10]. Our hypothesis is that a diet containing an abundance of processed food and low in micronutrient-rich plant foods can create physical symptoms of withdrawal when digestion ceases in between meals. Our contention is that during the catabolic phase of the digestion and refeeding cycle, when digestive activities cease, these withdrawal symptoms, misperceived as "hunger", develop from a diet that is inadequate or poor in micronutrients. We call these symptoms "toxic hunger". It is our clinical experience that such withdrawal symptoms drive overeating behavior and are a major factor leading to obesity. There is significant support for our observation and hypothesis, yet a relative lack of research on the determinants of overeating behavior and the hunger drive. A "dopaminic high" [11,12] from ingestion of high calorically concentrated sweets and fats has been documented and leads to subsequent craving of these foods. Very little human research has been done in this area.

In this investigation we were interested specifically in exploring the effect of a high nutrient density diet on participants' perceptions of hunger. We speculate that the discomfort of withdrawal from the toxins mobilized when one tries to refrain from consumption of pro-inflammatory processed foods and animal products may be also be a major contributor to compulsive eating and consequent obesity. Dietary micronutrients such as antioxidants and phytonutrients are required for the body to properly reduce the production and removal of metabolic waste products. In counseling patients to increase their micronutrient intake from greens and other nutrient-rich plant foods, our experience is that healthful eating is more effective for long-term weight control because it modifies and diminishes the sensations of withdrawal-related hunger, enabling overweight individuals to be more comfortable even while consuming substantially fewer calories.

In an attempt to further explore and validate our clinical observations, we conducted an internet-based survey of 768 individuals who had changed from the previous usual diet to a high nutrient density diet. The participants were recruited from a physician-run website of approximately 4000 subscribers designed to support the shift to a high nutrient density diet. In the survey we investigated the relationship between dietary micronutrient density and the symptoms of hunger.

## Subjects and Methods

### Population and Sample

We recruited by e-mail a convenience sample of 768 participants from a population of over 4000 subscribers to a website that provides education and support for people who wish to improve their eating habits to a high nutrient density diet. The website is hosted by a family physician specializing in nutrition. In addition to the website, most of the subscribers also have at least one of several books written by the physician on a healthy diet-style to maximize nutrition and weight reduction. The subscribers come from all parts of the United States and Canada, as well as a small number from Europe. About 65% of the subscribers to the website are female, with ages ranging from 35 to 65. Average household income of this population breaks down as follows: 27% $100-250K; 14% $85-100K; 10% $65-75K; and 8% $75-85K. Educational level of subscribers is: High School 16%; Vocational Technology credential 5%; Associate degree 11%; Bachelors 34%; Master 25%; Doctorate 9%. 71% are married, 17% single, 10% divorced and 1% are widowed. Information provided on the website includes the disease-protective and weight loss benefits of a diet rich in micronutrients from colorful vegetables, beans, seeds, nuts, fruits and whole grains and low (less than 10% of total calories) in processed foods and animal products.

Eligible participants were subscribers who indicated that they had made a change from their previous usual diet to a high nutrient density diet for at least one month. Consent was implied by clicking on a link to start the survey. The surveys were anonymous and were submitted via the Survey Monkey website[13]. Settings available on the Survey Monkey website were activated to block repeated submissions by participants.

### Instrument

A review of some of the commonly used instruments to assess hunger and eating behaviors such as the Three-Factor Eating Questionnaire and its earlier Eating Inventory [14,15] led us to conclude that these instruments do not assess in detail the actual symptoms of hunger. One tool developed by Friedman [14,16] does assess both the location and sensations of hunger. We did not find it to capture the specific issues that we were interested in examining and it would have been difficult to administer it online, so we developed our own internet-based pilot survey. The survey questions were divided into three conceptual categories: physical symptoms of hunger, emotional symptoms of hunger, and location of hunger. The items were revised per recommendation of other research team members through a step-wise process, to establish a preliminary content validity. The survey was then pilot tested with a group of twenty patients for ease of completion and clarity. After administration, internal consistency of the questions on the previous usual diet and the high nutrient density diet were analyzed using Cronbach's alpha. The alpha coefficient for the eight previous usual diet questions was .868 and for the eight high nutrient density diet questions was .851, suggesting that the items have relatively high internal consistency. Question 10 was omitted from the Cronbach analysis because it did not ask specifically about each kind of diet.

The survey questions referred to two types of diet: the participant's previous usual diet and the high nutrient density diet. Based on our clinical experience, we assumed that participants' previous usual diet received a majority of calories from processed, commercially prepared foods with added salt and sugars, oils, white flour as well as dairy and meats. Conversely, the high nutrient density diet is mostly unrefined, unprocessed plant food with minimal or no added salt, sugars, oils, and a minimal amount of animal products or no animal products. Access to the survey began with an informed consent question and the participant could only proceed by answering "yes". The remaining 13 questions were divided into 3 sections:

#### Section I: Dietary Questions (Questions 2-5)

These were four multiple choice questions about past and present diet style and eating habits including the degree and length of adherence to the high nutrient density diet. Two items served to measure the explanatory (independent) variables. Question 4 assessed **length **of adherence: "Approximately how long have you maintained the level of dietary change you indicated in the previous question?" The options were: less than 6 months, 6 to 12 months, more than 1 year, more than 5 years. Question 5 assessed **degree **of adherence: "Generally speaking, you adhere to a high nutrient density diet style". The options were: not very often, somewhat often, most of the time, all of the time.

#### Section II: Physical Sensations of Hunger (Questions 6 through 9)

This group of five Likert-scale questions asked about the participant's subjective symptoms and feelings of hunger including quality, and timing of symptoms.

#### Section III: Psychological, Mental and Emotional Aspects of Hunger (Questions 10 through 13)

The four Likert-scale questions in the final section pertained to how hunger affects the participant's emotions, thoughts and feelings.

#### Section IV: Location of Hunger

Question 14 presented a graphic of a human torso and asked participants to specify where on the graph they feel hunger.

### Ethics

The procedures we followed were in accordance with the ethical standards of and approval was obtained from the Institutional Review Board of the University of San Diego.

### Statistics

The purpose of our data analysis was (1) to compare across all questions differences in the experience of hunger in the high nutrient density diet versus the previous usual diet, (2) to determine if changes in the experience of hunger on the high nutrient density diet were greater as the length of time on and degree of adherence to the diet increased and (3) to determine if the location of hunger differed on the high nutrient density diet compared to the previous usual diet. Participants served as their own controls and answered the same questions about both diets. In essence, then, this was a pre-post test design. Due to the ordinal nature and non-normal distribution of the response variables, we chose to conduct non-parametric testing using SPSS statistical software (SPSS v18.0.2; Chicago: SPSS Inc.) per the following plan[17]:

1. Conduct the Wilcoxon test to compare the experience of hunger on the high nutrient density diet versus the previous usual diet.

2. Conduct the Spearman rho test to see if the experience of the discomforts of hunger with the high nutrient density diet was inversely correlated with the length of time on and adherence to the high nutrient density diet.

3. Perform the McNemar test to examine the relation between the type of diet and the location of hunger. Each possible location as indicated on a diagram was considered a dichotomous variable with choices "select" or "not select". The McNemar test provides the ability to conduct nonparametric testing comparing two groups when at least one variable is dichotomous [17].

## Results

### Descriptive statistics for the explanatory variables degree and length of adherence

Question 2 asked participants how familiar they are with the high nutrient density diet. Nearly 80% of participants indicated that they were "very familiar" with the diet. Question 3 asked participants to quantify their level of change to the high nutrient density diet. 76.5% of participants indicated that they adhere to the high nutrient density diet 75% to 100% of the time. Question 4 asked participants how long they had maintained their change to the high nutrient density diet. 23% had been on the high nutrient density diet less than 6 months, 16.9% between 6 and 12 months, 38.3% more than one year, and 12.1% more than 5 years. Question 5 asked participants to assess how often they observe the high nutrient density diet. 1.6% indicated not very often, 10.4% indicated somewhat often, 54,8% indicated most of the time, and 24.3% indicated all of the time.

As indicated above, questions 4 and 5 were used to measure the explanatory variables **length **and **degree **of adherence, against which responses to the questions related to the experience of hunger on the high nutrient density diet were compared.

### Physical experience of hunger on high nutrient density diet vs. previous usual diet

**Figure 1:**
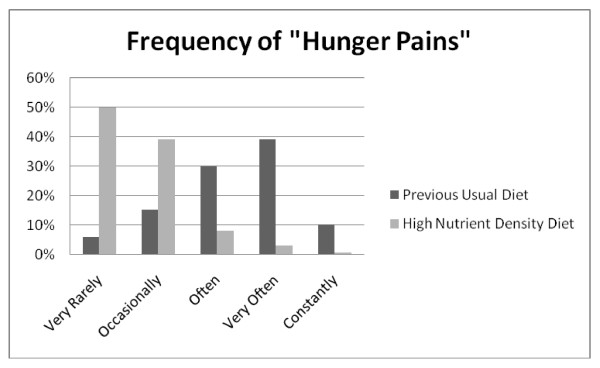
**Q6: HUNGER PAINS**. Hunger pains were experienced less often when on the high nutrient density diet compared to the previous usual diet, *Z *= -18.835, p < 0.001.

**Figure 2:**
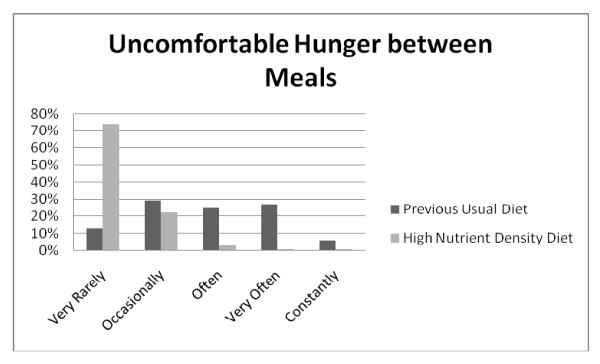
**Q7: BETWEEN MEALS**. Hunger symptoms between meals were experienced less often when on the high nutrient density diet compared to the previous usual diet, *Z = -18.927*, p < 0.001.

**Figure 3:**
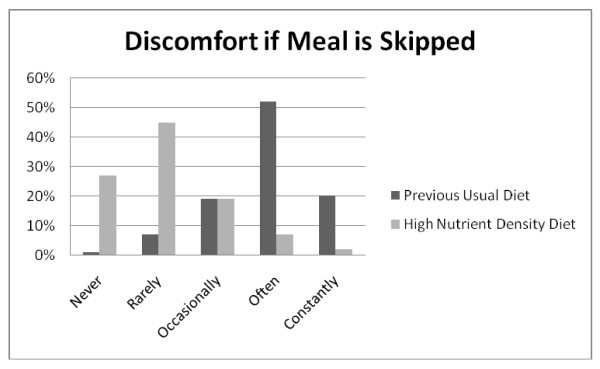
**Q8: SKIPPED MEALS**. Hunger symptoms with skipped meals were experienced less often when on the high nutrient density diet compared to the previous usual diet, *Z = -19.513*, p < 0.001.

**Figure 4:**
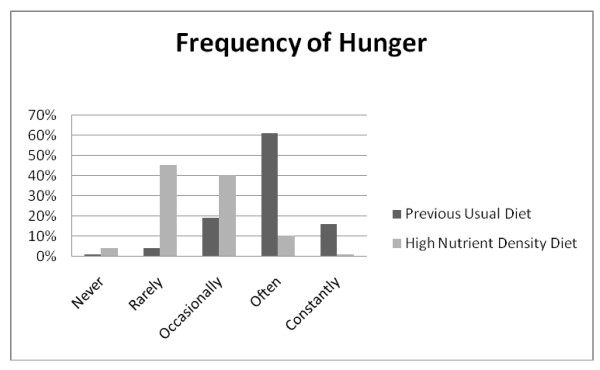
**Q9: HUNGER FREQUENCY**. Hunger was experienced less often when on the high nutrient density diet compared to the previous usual diet, *Z = -18.2527*, p < 0.001.

**Figure 5:**
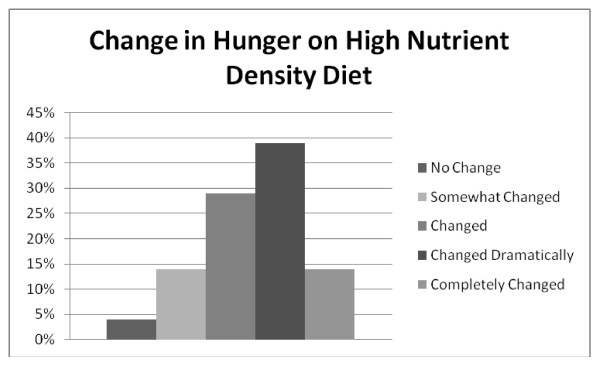
**Q10: CHANGE IN HUNGER EXPERIENCE**. Nearly 80% of respondents reported that their experience of hunger had changed since starting the high nutrient density diet, with 51% reporting a dramatic or complete change in their experience of hunger.

A Wilcoxon Signed-ranks test indicated significant differences between the physical experience of hunger on the previous usual diet versus the high nutrient density diet for participants across all questions on this issue. See figures 1, 2, 3, and 4 showing the results for this set of questions. Figure 5 shows the responses to the general question of how much the experience of hunger has changed since starting on the high nutrient density diet. Nearly 80% of respondents reported that it had changed, with 51% reporting a dramatic or complete change in their experience of hunger.

### Emotional experience of hunger on high nutrient density diet vs. previous usual diet

**Figure 6:**
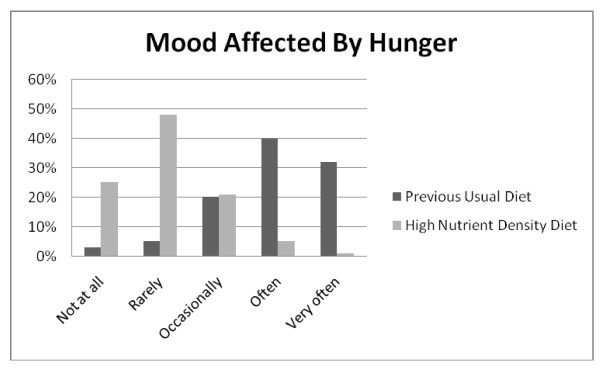
**Q11: MOOD FLUCTUATIONS**. Mood was less affected by hunger on the high nutrient density diet compared to the previous usual diet, *Z = -19.165*, p < 0.001.

**Figure 7:**
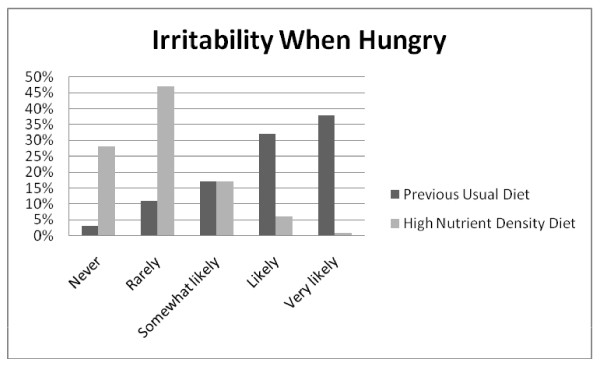
**Q12: IRRITABILITY**. Irritability when hungry was less likely to be experienced on the high nutrient density diet compared to the previous usual diet, *Z = -18.937*, p < 0.001.

**Figure 8:**
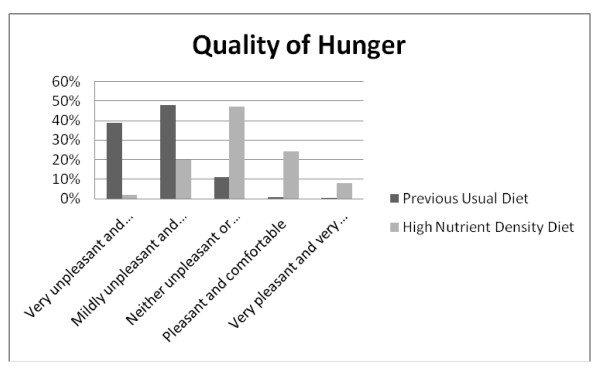
**Q13: UNPLEASANTNESS**. Hunger was described as being unpleasant less often when on the high nutrient density diet compared to the previous usual diet, *Z = -18.368*, p < 0.001.

A Wilcoxon Signed-ranks test indicated significant differences between the emotional experience of hunger on the previous usual diet versus the high nutrient density diet for participants across all questions on this issue. See figures 6, 7, and 8.

### Location of hunger on high nutrient density diet vs. previous usual diet

**Figure 9:**
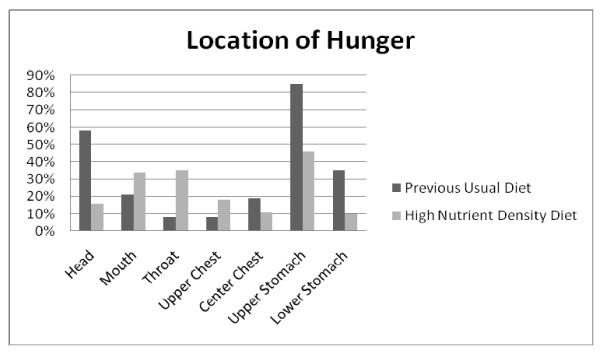
**Question 14: LOCATION OF HUNGER**. The high nutrient density diet was associated more often with hunger in the mouth, throat, chest and upper stomach; the previous usual diet was associated more often with hunger in the head, and upper/lower stomach. Using the McNemar test, differences in choice of location of hunger between the two diets were highly significant (p < 0.001) across all locations tested.

Each of the selections for location of hunger on the reference chart was dichotomized to 'selected' versus 'not selected'. Figure 9 illustrates the percentage of participants who chose each location in reference to the previous usual diet and the high nutrient density diet. For the previous usual diet the largest percentage selected was for "upper abdomen/mid stomach" (69.9%, n = 489) followed by "head" (47.4%, n = 332). The lowest was for 'throat' (6.4%, n = 45). For the high nutrient density diet, the largest percentage selected was for "upper abdomen/mid stomach" (39.4%, n = 276) followed by "throat" (29.9%, n = 209). The lowest was for "lower stomach/upper intestine" (8.6%, n = 60). Using the McNemar test, differences in choice of location of hunger between the two diets were highly significant (p < 0.001) across all locations tested.

### Correlations between length on and adherence to high nutrient density diet and experience of hunger

Tables 1 and 2 summarize the Spearman rho correlation coefficients comparing the physical and emotional experience of hunger while on the high nutrient density diet to length and degree of adherence to the diet. Significant inverse correlations between adherence and all physical and emotional discomforts of hunger were present across all questions; frequency of hunger was not significantly correlated with either length or adherence. However, length of time on the high nutrient density diet was significantly inversely correlated with the frequency of hunger pains in the stomach and discomfort with skipped meals.

**Table 1:** Spearman's rho correlations between physical experience of hunger and length of time on and degree of adherence to the high nutrient density diet.

**6B. Frequency of hunger pains on high nutrient density diet**	**Length**	**Adherence**
Correlation coefficient *r*	-.116**	-.195**
Significance (2-tailed)	.003	.000
N	651	649
**7B. Discomfort between meals on high nutrient density diet**		
Correlation coefficient *r*	-.046	-.199**
Significance (2-tailed)	.237	.000
N	650	648
**8B. Discomfort if meal is skipped on high nutrient density diet**		
Correlation coefficient *r*	-.140**	-.258**
Significance (2-tailed)	.000	.000
N	642	640
**9B. Frequency of hunger on high nutrient density diet**		
Correlation coefficient *r*	-.059	-.058
Significance (2-tailed)	.134	.141
N	647	645
**10. How much hunger has changed on high nutrient density diet**		
Correlation coefficient *r*	.016	.266**
Significance (2-tailed)	.696	.000
N	618	616

**significant correlations at p < 0.05

**Table 2:** Spearman's rho correlations between emotional experience of hunger and length of time on and degree of adherence to the high nutrient density diet.

**11B. Mood affected by hunger on high nutrient density diet**	**Length**	**Adherence**
Correlation coefficient *r*	-.042	-.208**
Significance (2-tailed)	.302	.000
N	615	613
**12B. Irritable when hungry on high nutrient density diet**		
Correlation coefficient *r*	-.036	-.173**
Significance (2-tailed)	.373	.000
N	615	614
**13B. Hunger is less unpleasant on high nutrient density diet**		
Correlation coefficient *r*	.054	.169**
Significance (2-tailed)	.182	.000
N	614	612

**significant correlations at p < 0.05.

## Discussion

This study provides important insights into hunger in a society characterized by over-consumption of processed food with an excess of calories and deficiency of micronutrients. Such hunger creates a cycle of overeating leading to obesity and is an obstacle for those who attempt to establish a healthy eating pattern and normal BMI. We found highly significant differences in the experience of hunger on the high nutrient density diet compared to the previous usual diet in a large sample of people who had made the shift to a diet high in micronutrients and lower in calories. The uncomfortable physical and emotional symptoms of hunger were much less prevalent after a change to the high nutrient density diet was made. We also observed a "dose response" that was strongly correlated with the degree of adherence to the high nutrient density diet. Our findings reveal that those who are able to make the change to a high nutrient density diet experience uncomfortable sensations of hunger less often than they experienced on their previous usual diet. In this survey of 768 participants, over 75% indicated that they observe the high nutrient density diet most or all of the time. Participants who adhered to the high nutrient density diet overall found hunger to be an uncomfortable experience less often; this may explain the previously reported high levels of compliance and successful weight loss [18] with the high nutrient density diet. Their hunger was less often characterized by classic withdrawal symptoms such as headaches, tremors, stomach cramps, and mood changes. Rather, it was more often felt as a throat sensation that was easily tolerated.

**Figure 10:**
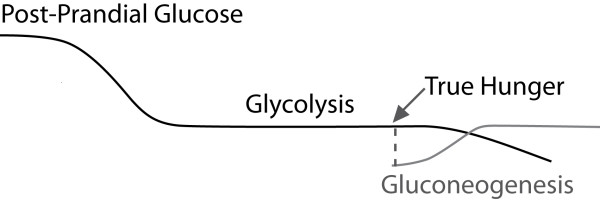
**THE GLUCOSE RESPONSE CURVE**. True hunger occurs when glycogen stores are depleted, so that gluconeogenesis can be avoided.

As soon as the intake, digestion and assimilation of food is complete, the catabolic utilization of glycogen reserves and fatty acid stores begins. Hunger normally increases in intensity as glycogen stores are diminishing toward the end of glycolysis, and should not occur at the start of the catabolic phase when glycolysis begins (see Figure 10). It is our contention that uncomfortable symptoms that drive overeating behaviors early in the catabolic phase should be recognized as withdrawal symptoms from a sub-optimal diet and not true hunger. After the completion of digestive activity, during catabolism, the mobilization and elimination of cellular waste products are heightened, thus precipitating symptoms commonly thought to be hunger. In contrast, true hunger occurs much later when glycogen stores near completion, preventing gluconeogenesis. Gluconeogenisis is the utilization of muscle tissue for needed glucose once glycogen stores have been depleted. True hunger protects lean body mass, but does not fuel fat deposition. It exists to protect lean body mass from utilization as an energy source.

Recent research on the physiology of metabolism provides a plausible explanation for our findings. When a diet is low in dietary antioxidants, phytochemicals and other micronutrients, intra-cellular waste products such as free radicals, advanced glycation end products, lipofuscin, lipid A2E, and others accumulate [9,19]. Other studies have demonstrated an adverse impact of low-micronutrient foods containing higher amounts of simple carbohydrates, fats and animal products on levels of inflammatory markers, metabolic by-products and oxidative stress in the body [20,21]. It is well established in the scientific literature that these substances contribute to disease [22-25], and can be associated with typical withdrawal symptoms, including headaches [26,27]. Heightened elimination of these waste products may create symptoms that can be experienced similarly to withdrawal from drug addiction [28]. In the absence of an adequate intake of phytochemicals and other micronutrients, cellular detoxification is impaired [29] which elevates cellular free radical activity, priming the body with more substrate to induce withdrawal symptoms when digestion ceases. Our theory is that these uncomfortable symptoms, relieved by eating which halts catabolism and arrests the detoxification process, are widely misperceived as hunger. In a society with an abundance of fast food and high rates of obesity, commonly experienced sensations of hunger may actually be symptoms of withdrawal from a diet that is inadequate in micronutrients. Such a diet creates an excess of pro-inflammatory metabolic waste products as well as an addiction syndrome. There is growing evidence that food addiction is a clinical pathological condition [30-43]. Our hypothesis, supported by this pilot study, is that this addiction is caused by withdrawal symptoms misread as hunger from pro-inflammatory foods and can be mitigated by consumption of a diet high in anti-inflammatory micronutrients found in vegetables and other micronutrient-rich plant foods.

Evidence suggests that overweight individuals build up more inflammatory markers and oxidative stress when fed a low nutrient meal compared to normal weight individuals [20,21]. The heightened inflammatory potential in those with a tendency for obesity is marked by increasing levels of lipid peroxidase and malondialdehyde and reduced activation of hepatic detoxification enzymes [44]. This is supportive of our experience that people prone to obesity get more withdrawal/hunger symptoms, preventing them from being comfortable in the non-digestive (catabolic) stage where breakdown and mobilization of toxins is enhanced. The resulting uncomfortable symptoms drive them to eat again and over-consume calories. It is a vicious cycle promoting continuous (anabolic) digestion, frequent feedings and increased intake of calories. Chronically overweight people in the typical American food environment feel "normal" only by eating too frequently or by eating a heavy meal, so that the anabolic process of digestion and assimilation continues right up to the beginning of the next meal. In both cases, as our overweight patients report, excess calories are needed in order to feel normal. A review of research on companion animals suggested that the introduction of specific micronutrients positively influenced the health status of animals whose natural detoxification systems were compromised, and reduced the accumulation of inflammatory markers [29]. This may explain why those on the high nutrient density diet were able to go for longer periods without feeling "hunger" symptoms.

There exists only a small body of previous research exploring the relationship between the type of foods ingested and the intensity and/or frequency of hunger. One theory that has been investigated is the glucostatic theory which links dynamic changes in blood glucose with appetitive sensations [45-48]. Several studies have explored the relation between the glycemic index or fiber content of food and satiety, whereas others have examined whether the type or amount of fatty acids, sugars or protein in the diet affect the sense of hunger [49-62]. Results have been inconsistent. This may be due to the unknown variable of micronutrient intake in these studies. Some studies have documented a decrease in appetite with ingestion of greater amounts of fiber and/or micronutrients [49,52,56]. Recently, a Canadian study found that fasting and postprandial appetite ratings were reduced in women who were supplemented with multivitamins and minerals [63].

The findings of this study are particularly significant given the nature of the diet we studied. Highly significant reductions in blood pressure, LDL cholesterol, fasting glucose and body weight have been reported in persons who have made the change to a high micronutrient diet [18]. Further, there is a vast body of research documenting the protective benefit of a micronutrient-rich diet against cancer and cardiovascular disease [1,8,10,24,25,64-77]. If clinicians can assure their patients with confidence that they will not experience uncomfortable sensations of hunger after the "detoxification" stage is over, they can keep their patients motivated to withstand the withdrawal symptoms they experience early in the dietary transition. The outcome will be not only substantial and sustainable weight loss, but prevention of many major chronic diseases in our patients. Our hypothesis clearly requires further study and testing, but this preliminary study justifies additional investigations into this interesting and significant issue.

We must acknowledge the limitations of this study, including the fact that this was a retrospective, non-controlled study. The instrument we used has not been validated on large or diverse populations, although we did establish preliminary internal consistency and content validity. We recognize that participants were self-selected and may have been biased in their responses by exposure to the information on the website and resources to which they all subscribed. There are discussions of "toxic hunger" versus "true hunger" in the written and web-based materials that participants had access to. Participants were, however, assured of the anonymity of their responses in the introduction to the survey, and the survey responses were received from the Survey Monkey website without any identifying information, including no inclusion of email addresses of those who completed the surveys. It will be important to see if this dramatic shift in hunger perception would be found in populations not exposed to "leading" messages in future studies. We also did not assess the actual diet that each participant typically maintained prior to changing to the high nutrient density diet, nor did we validate the self reports of degree of compliance to the high nutrient density diet. Future studies should include food diaries and measures of biomarkers to quantify these variables more precisely.

However, given these limitations, the number of participants and highly significant test statistics provide leads for future studies that are better controlled and prospective in design and some important clinical insights. Further studies should explore the physiological and neurohormonal correlates of "toxic hunger" and of "true hunger", including measures of oxidative stress and ghrelin levels in people who adhere to the high nutrient density diet and the previous usual diet. It would also be helpful to examine how long the typical "withdrawal phase" from the previous usual diet lasts as people shift to the high nutrient density diet. This information would be valuable in our clinical efforts to support those who are making the change to healthier eating patterns.

## Conclusions

We found significant differences in the symptoms, location and unpleasantness of hunger on the high nutrient density diet compared to the participants' previous usual diet in a large sample of people who had made the shift to a diet high in micronutrients and lower in calories. Hunger is one of the major impediments to successful weight loss. Our findings suggest that it is not simply the caloric content, but more importantly, the micronutrient density of a diet that influences the experience of hunger. It appears that a high nutrient density diet, after an initial phase of adjustment during which a person experiences "toxic hunger" due to withdrawal from pro-inflammatory foods, can result in a sustainable eating pattern that leads to weight loss and improved health. Further studies are needed to confirm these preliminary findings that may have important implications in the global effort to control rates of obesity and related chronic diseases.

## Competing interests

JF reports that he is the author of the book and website used to guide participants who adhered to the diet discussed in this study. BS, DG and SA declare that they have no competing interests.

## Authors' contributions

JF participated in the conception and design of the study and survey instrument, and writing of the manuscript. BS participated in the conception and design of the study, summarizing and reporting of statistics and writing of the manuscript. DG conducted the statistical analyses of the study. SA participated in the design and administration of the survey instrument and the collection of data. All authors read and approved the final manuscript.
